# Properdin inhibition ameliorates hepatic ischemia/reperfusion injury without interfering with liver regeneration in mice

**DOI:** 10.3389/fimmu.2023.1174243

**Published:** 2023-08-16

**Authors:** Jiro Kusakabe, Koichiro Hata, Tetsuya Tajima, Hidetaka Miyauchi, Xiangdong Zhao, Shoichi Kageyama, Tatsuaki Tsuruyama, Etsuro Hatano

**Affiliations:** ^1^ Department of Surgery, Division of Hepato-Biliary-Pancreatic Surgery and Transplantation, Graduate School of Medicine, Kyoto University, Kyoto, Japan; ^2^ Center for Anatomical, Pathological, and Forensic Medical Research, Graduate School of Medicine, Kyoto University, Kyoto, Japan

**Keywords:** hepatic ischemia/reperfusion injury, liver transplantation, hepatectomy, complement, alternative pathway, properdin

## Abstract

Hepatic ischemia/reperfusion injury (IRI) often causes serious complications in liver surgeries, including transplantation. Complement activation seems to be involved in hepatic IRI; however, no complement-targeted intervention has been clinically applied. We investigated the therapeutic potential of Properdin-targeted complement regulation in hepatic IRI. Male wild-type mice (B10D2/nSn) were exposed to 90-minute partial hepatic IRI to the left and median lobes with either monoclonal anti-Properdin-antibody (Ab) or control-immunoglobulin (IgG) administration. Since the complement system is closely involved in liver regeneration, the influence of anti-Properdin-Ab on liver regeneration was also evaluated in a mouse model of 70% partial hepatectomy. Anti-Properdin-Ab significantly reduced serum transaminases and histopathological damages at 2 and 6 hours after reperfusion (*P <*0.001, respectively). These improvements at 2 hours was accompanied by significant reductions in CD41+ platelet aggregation (*P* =0.010) and ssDNA+ cells (*P <*0.001), indicating significant amelioration in hepatic microcirculation and apoptosis, respectively. Characteristically, F4/80+ cells representing macrophages, mainly Kupffer cells, were maintained by anti-Properdin-Ab (*P <*0.001). Western blot showed decreased phosphorylation of only Erk1/2 among MAPKs (*P* =0.004). After 6 hours of reperfusion, anti-Properdin-Ab significantly attenuated the release of HMGB-1, which provokes the release of proinflammatory cytokines/chemokines (*P* =0.002). Infiltration of CD11b+ and Ly6-G+ cells, representing infiltrating macrophages and neutrophils, respectively, were significantly alleviated by anti-Properdin-Ab (both *P <*0.001). Notably, anti-Properdin-Ab did not affect remnant liver weight and BrdU+ cells at 48 hours after 70% partial hepatectomy (*P* =0.13 and 0.31, respectively). In conclusion, Properdin inhibition significantly ameliorates hepatic IRI without interfering with liver regeneration.

## 1 Introduction

Hepatic ischemia/reperfusion (IR) injury (IRI) arises from the interruption of liver blood flow and its subsequent restoration, and is characterized by sterile inflammation and hepatocyte death (1). Hepatic IRI is a risk factor for liver failure after extensive liver resection. In liver transplantation, it is associated with graft dysfunction and acute/chronic rejection (2, 3). In addition, extended-criteria donor organs are vulnerable to hepatic IRI. To improve the outcomes of liver surgery, novel therapeutic interventions need to be developed for the deleterious condition.

The complement system is an important mediator in the innate and adaptive immune reactions (4). The system has recently been attracting considerable attention, since eculizumab, a humanized monoclonal antibody (mAb) to C5, has demonstrated remarkable therapeutic efficacy against various intractable diseases, including paroxysmal nocturnal hemoglobinuria, atypical hemolytic uremic syndrome, and refractory generalized myasthenia gravis (5). The complement system is also closely involved in hepatic IRI (6, 7). Various approaches have reportedly been effective to attenuate hepatic IRI, including cobra venom factor, C1-inhibitor, soluble complement receptor-1 (sCR-1), C5a, and C5b-9 inhibitors (6, 8–12). However, complement-targeted therapies for hepatic IRI have yet to be introduced into clinical practice.

Properdin, a plasma glycoprotein, is known to be the only component that positively regulates the alternative pathway (AP) of the complement system (13–15). The AP amplifies all complement activity, accounting for approximately 80% of terminal pathway activity (13). Properdin accelerates AP activation by binding to and stabilizing the C3- and C5-convertases, thereby upregulating their activity 5- to 10-fold to cleave C3 to C3a and C3b, and C5 to C5a and C5b, respectively (13, 15). *In vivo* murine studies reported that Properdin inhibition may be a favorable intervention for inflammatory diseases such as arthritis, asthma, and renal IRI (16–18). Furthermore, efficacy of anti-Properdin-antibody has been evaluated in a phase two trial for age-related macular degeneration (5). Collectively, Properdin inhibition has a remarkable impact on both C3 and terminal pathway activities, and we hypothesized that it would contribute substantially to alleviating hepatic IRI.

The present study thus investigated the therapeutic potential of Properdin regulation in a murine model of hepatic IRI. On the other hand, there is a concern that complement inhibition may negatively affect liver regeneration after extensive hepatectomy or partial liver transplantation (19). Accordingly, we also assessed the influence of Properdin inhibition on liver regeneration in a mouse model of partial hepatectomy.

## 2 Materials and methods

### 2.1 Animals

Male wild-type, B10D2nSn-Slc mice (8–10 weeks, 20–25 g) were purchased from Japan SLC, Inc. (Hamamatsu, Japan). All animals were housed in specific pathogen-free conditions in a temperature- and humidity-controlled environment with a 12-hour light-dark cycle, and were provided *ad libitum* access to tap water and standard chow pellets. All animals received humane care in accordance with the Animal Research: Reporting of *In Vivo* Experiments (ARRIVE) guidelines. All experimental protocols were approved by the Animal Research Committee of Kyoto University (MedKyo-17546 and -18193). All authors had access to the study data and had reviewed and approved the final manuscript.

### 2.2 Liver IRI model

**Figure 1:**
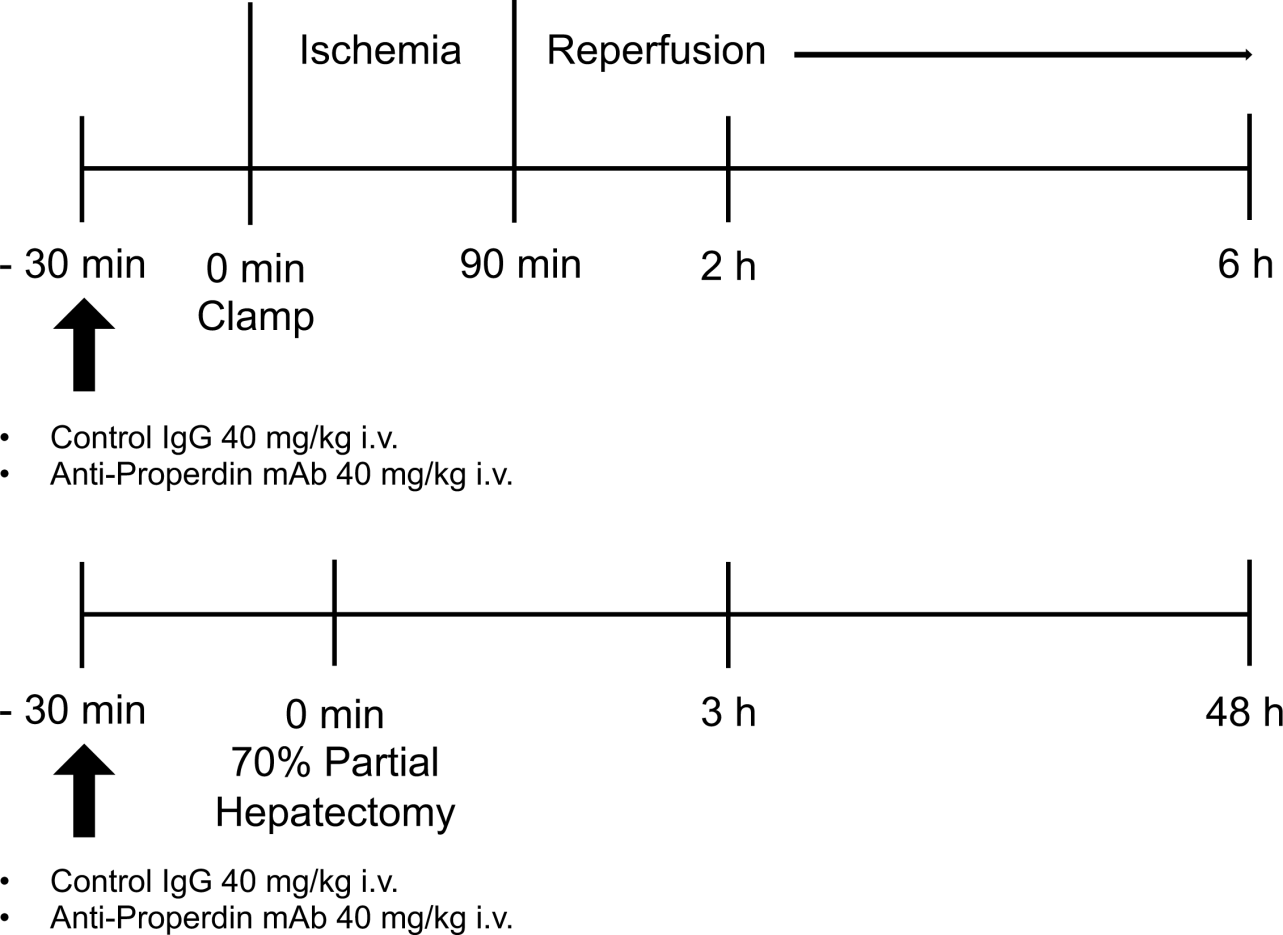
Schematic illustration of the experimental design. In the hepatic IR model, male WT mice, B10D2nSn-Slc, were exposed to 70% partial hepatic ischemia of the left and median lobes for 90 minutes, followed by reperfusion. Liver tissues and blood were sampled at 2 and 6 hours after reperfusion (*n* = 6 mice/group at each time point). In the 70% partial hepatectomy model, liver tissues and blood samples were obtained at 3 and 48 hours after the procedure (*n* = 8 mice/group at each time point). Either IgG1 isotype control (clone MOPC-21) or anti-Properdin-Ab was administered 30 minutes before ischemia or surgery in each model. IR, ischemia/reperfusion; WT, wild-type; IgG, immunoglobulin; Ab, antibody.

The established mouse model of partial warm hepatic IRI was used (20) (**Figure 1**). Mice were anesthetized under isoflurane. We used an atraumatic clip (AS-1; Natsume Seisakujo Co., Ltd., Tokyo, Japan) to interrupt both arterial and portal-venous supply to the left and middle liver lobes. After 90 minutes of ischemia, reperfusion was initiated by removing the clamp (**Figure 1**). Mice were given an intravenous injection of anti-Properdin-Ab, provided by Alexion Pharmaceuticals (Cheshire, CT) at a dose of 40 mg/kg 30 min prior to ischemic insult (21); they were then humanely sacrificed at 2 and 6 hours after reperfusion (*n* = 6 mice/group at each time point). Mice were pretreated with the same dose of control immunoglobulin (mouse IgG-1 isotype control, clone MOPC-21, BioXCell, NH). Sham-operated mice underwent the same procedure, but without any vascular occlusion.

### 2.3 70% partial hepatectomy model

Under general anesthesia with isoflurane, 70% partial hepatectomy was performed as previously described (22, 23), with resection of the median and left lateral liver lobes (**Figure 1**). Mice were given an intravenous injection of anti-Properdin-Ab at a dose of 40 mg/kg 30 min prior to ischemic insult (21); they were then humanely sacrificed at 3 and 48 hours after surgery (*n* = 8 mice/group at each time point). Mice were pretreated with the same dose of control IgG. Sham-operated mice underwent the same procedure, but without liver resection. Increase in liver weight (%) was calculated as follows: [C−(B−A)]/(B−A)*100, where A is the weight of the resected liver (approximately 70% of total liver), B is the estimated total liver weight based on the weight of the resected liver (B = A/0.7), and C is the final liver weight at the time of sacrifice.

### 2.4 Complement hemolytic assay

Functional activity of the alternative pathway in mouse sera was measured by the standard method to assess its ability to lyse rabbit erythrocytes (24, 25). Briefly, 2mM MgCl_2_ and 10mM EGTA were added to rabbit erythrocytes diluted in GVBS (B100, CompTech, Tyler, TX). The dilution was washed with centrifugation and blood cell pellet was resuspended. Each sample and the dilution were added to a 96-well plate, and then incubated at 37°C for 30 min. After centrifugation, the supernatant was transferred to a microtiter plate and then read at OD415 using a microplate reader.

### 2.5 Hepatocellular damage

Serum alanine aminotransferase (ALT) levels in peripheral blood, an indicator of hepatocellular injury, were measured using a standard spectrophotometric method with an automated clinical analyzer (JCA-BM9030; JEOL, Ltd., Tokyo, Japan).

### 2.6 Histology

Paraffin-embedded liver tissue specimens (thickness of 4 μm) were stained with hematoxylin & eosin. The severity of hepatic IRI (necrosis, sinusoidal congestion, and centrilobular ballooning) was blindly graded by two independent pathologists, according to the modified Suzuki criteria on a scale of 0 to 4 (26).

### 2.7 Enzyme-linked immunosorbent assay

Serum high-mobility group box-1 (HMGB-1) levels were measured with HMGB-1 ELISA Kit II (Shino-Test, Tokyo, Japan) according to the manufacturer’s protocol. Serum interleukin (IL)-6 and tumor necrosis factor (TNF)-α levels were measured using Mouse Quantikine ELISA Kits (R&D Systems, Minneapolis, MN, USA) in accordance with the manufacturer’s instructions.

### 2.8 Quantitative reverse-transcription polymerase chain reaction

Proinflammatory cytokines (*Il1β, Il6*, and *Tnf*) and chemokines (*Cxcl1* and *Cxcl2*) in liver tissues with or without Properdin regulation were analyzed by qRT-PCR. Total RNA was extracted from liver tissues using an RNeasy Kit (Qiagen, Venlo, Netherlands) and complementary DNA was prepared using an Omniscript RT kit (Qiagen). qRT-PCR was performed using the StepOnePlus Real-Time PCR System (Life Technologies, Tokyo, Japan). The primers used to amplify specific gene fragments are listed in **Supplemental Table 1**. Target gene expression was calculated by the ratio to the housekeeping gene, glyceraldehyde 3-phosphate dehydrogenase (*Gapdh*). The data were represented as fold differences by the 2^-ΔΔCt^ method, where ΔCt = Ct_target gene_ - Ct_GAPDH_ and ΔΔCt = ΔCt_induced_ - ΔCt_reference_, with naive mice as a reference.

### 2.9 Immunohistochemistry

After deparaffinization of liver sections, antigen was retrieved with citrate buffer (10 mM, pH 6.0). After blocking with Protein Block Serum-Free (X0909, DAKO, Tokyo, Japan), the primary antibodies were applied to specimens (**Supplemental Table 2**). In light microscopy, sections were treated with biotinylated rabbit anti-rat IgG and goat anti-rabbit IgG (1:300). After incubation, immunoperoxidase (VECTASTAIN Elite ABC Kit, Vector Labs, Burlingame, CA, USA) was applied and then visualized using 3,3’-diaminobenzidine tetrahydrochloride (DAB, Sigma-Aldrich, St. Louis, MO, USA) solution with hematoxylin counterstaining. Exceptionally, Avidin-biotin-alkaliphosphatase complex (VECTASTAIN ABC-AP Standard Kit, Vector Labs, Burlingame, CA, USA) and Fast red (Fast Red II Substrate kit, Nichirei Biosciences Inc., Tokyo, Japan) were used for visualization for 8-OHdG staining instead of avidin-peroxidase complex and DAB because endogenous peroxidase activity was not blocked by H_2_O_2,_ which produces hydroxiradical, converting deoxyguanosine (dG) into 8-OHdG. In fluorescence microscopy, sections were treated with Alexa 488-conjugated goat anti-rat IgG (1:500) and covered with Vectashield mounting medium containing 4,6-diamidino-2-phenylindole (Vector Laboratories, Burlingame, CA, USA). Sections were observed with a BZ-9000 fluorescence microscope (Keyence, Osaka, Japan). Positive cells were enumerated blindly at 10 high-power fields/section (× 400). Negative controls were prepared by incubation with normal rat-IgG or rabbit-IgG (sc-2026, -2027; Santa Cruz Biotechnology, Santa Cruz, CA, USA) instead of the first Abs. The CD41-positive area was quantified using ImageJ software (National Institutes of Health, Bethesda, MD, USA).

### 2.10 Western blot

Liver tissues were homogenized in radioimmunoprecipitation buffer (Thermo Fisher Scientific Inc., Waltham, MA, USA), soluble protein lysates (30 μg/sample) were subjected to 12%-gradient sodium dodecyl sulfate polyacrylamide gel electrophoresis, and the protein bands were transferred onto polyvinylidene difluoride (PVDF) membranes (Bio-Rad, Hercules, CA, USA). After blocking the membranes with 5% skim milk, they were probed with unconjugated primary antibodies (**Supplemental Table 3**) in the dilution buffer (0.5% skim-milk in Tris-buffered saline-Tween 20) with overnight agitation at 4°C. After washing, the membranes were reacted with the horseradish peroxidase-conjugated secondary antibody (P0448; DAKO, Santa Clara, CA). Chemiluminescence was detected using ImmunoStar Zeta (WAKO Pure Chemical Industries, Ltd., Osaka, Japan), and visualized with a charge-coupled device camera (EZ-capture, Atto Corporation, Tokyo, Japan). Band intensity was quantified with ImageJ Software.

### 2.11 Statistical analysis

All data are expressed as means ± the standard error of the mean (SEM). Differences between the experimental groups were analyzed using Student’s t test or One-way ANOVA with Bonferroni *post-hoc* test as appropriate. *P <*0.05 was considered to be significant. All calculations were performed with Prism 7 (Graph Pad Software, Inc., San Diego, CA).

## 3 Results

### 3.1 Suppression of alternative pathway by anti-Properdin-Ab in hepatic IRI

**Figure 2:**
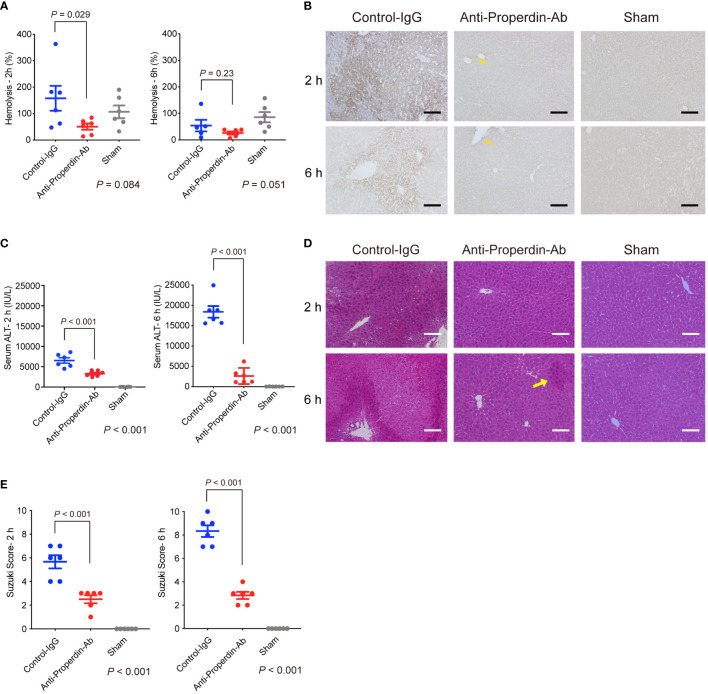
Hemolytic/complement activity and liver damage in hepatic IRI model. **(A)** Serum hemolytic activity was measured at 2 and 6 hours after reperfusion to assess the functional activity of the alternative pathway. It was significantly reduced by anti-Properdin-Ab compared with by control IgG at 2 h after reperfusion. (*n* = 6 mice/group at each time point). Data from sham controls are shown as a reference. **(B)** Representative liver sections for immunohistochemical staining for C3 at 2 h and 6 h after reperfusion (*n* = 6 mice/group, magnification, ×200). Scale bars indicate 100 μm. C3 deposition was observed diffusely in the control group, whereas it was observed only in some hepatocytes around the central veins (zone 3) (yellow arrows) at each time point. **(C)** Serum ALT was significantly lower in anti-Properdin-Ab group than in the control group at 2 and 6 hours after reperfusion (*n* = 6 mice/group at each time point). Data from sham controls are shown as a reference. **(D)** Representative hematoxylin-eosin-stained liver tissue sections at 2 and 6 hours after reperfusion (magnification, ×200, Scale bar: 100 μm). Liver tissue damages, e.g., lobular edema, congestion, ballooning, and hepatocellular necrosis, were diffusely observed in the control group at both time points, whereas they were sporadically seen in anti-Properdin-Ab group (yellow arrow). **(E)** Histopathological grading (*n* = 6 mice/group at each time point). Consistent with ALT release, tissue damage was significantly alleviated upon anti-Properdin-Ab treatment. IgG, immunoglobulin; Ab, antibody; IRI, ischemia/reperfusion injury.

Serum hemolytic activity was assessed at 2 and 6 hours after reperfusion. As shown in **Figure 2A**, anti-Properdin-Ab significantly decreased hemolytic activity compared with control immunoglobulin (IgG) at 2 hours after reperfusion. In liver tissues after hepatic IRI, diffuse and dense C3-deposition was observed in the control group, which was substantially attenuated by anti-Properdin-Ab and only sporadically observed around the central veins (zone 3) at 2 and 6 hours after reperfusion (**Figure 2B**).

### 3.2 Transaminase release and histopathological analyses

Serum ALT was significantly lower in anti-Properdin-Ab group than in the control group at 2 and 6 hours after reperfusion (**Figure 2C**). Consistently, liver tissue damages, e.g., lobular edema, congestion, ballooning, and hepatocellular necrosis, were significantly ameliorated by anti-Properdin-Ab (Suzuki’s histological score: P <0.001 at 2 and 6 hours after reperfusion, **Figures 2D, E**).

### 3.3 HMGB-1 release

**Figure 3:**
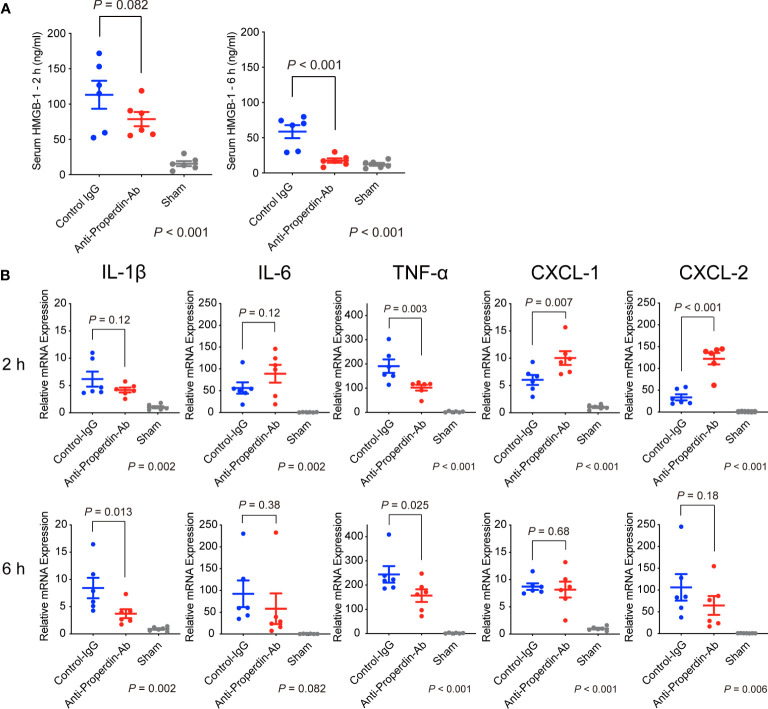
HMGB-1 release and proinflammatory cytokines/chemokines. **(A)** Serum HMGB-1 concentrations were higher at 2 h after reperfusion than at 6 hours in the control group. Anti-Properdin-Ab significantly attenuated HMGB-1 release at 6 h (*n* = 6 mice/group). Data from sham controls are shown as a reference. **(B)** Quantitative Reverse-Transcription Polymerase Chain Reaction analysis of expression of proinflammatory cytokines (*Il1β*, *Il6*, and *Tnf*) and chemokines (*Cxcl1* and *Cxcl2*). Although no consistent trends were observed at 2 hours after reperfusion, anti-Properdin-Ab significantly reduced the expression of TNF-α (**Figure 3B**). The expressions of CXCL-1 and -2 were significantly higher in the anti-Properdin-Ab group. At 6 hours, anti-Properdin-Ab significantly decreased the expressions of IL-1β and TNF-α. Data from sham controls are shown as a reference. Gene expression levels were normalized to those of *Gapdh* (*n* = 6 mice/group). HMGB‐1, high‐mobility group box 1 protein; IgG, immunoglobulin; Ab, antibody.

HMGB-1 is a nuclear factor that functions as an early mediator of inflammation and liver damage in hepatic IRI (27). When released extracellularly, it raises massive release of proinflammatory cytokines/chemokines by interacting with soluble molecules such as Toll-like receptor-4. To comprehensively evaluate liver damage and assess the impact of HMGB-1 release on subsequent inflammatory responses, serum levels of HMGB-1 were determined using ELISA. Serum HMGB-1 concentrations were higher at 2 hours after reperfusion than at 6 hours in the control group (**Figure 3A**). Anti-Properdin-Ab significantly attenuated HMGB-1 release at 6 hours (*P* =0.002).

### 3.4 Pro-inflammatory cytokines and chemokines

Proinflammatory cytokines (IL-1β, IL-6, and TNF-α) play pivotal roles in the pathogenesis of hepatic IRI (28). CXC chemokines, including CXCL-1 and CXCL- 2, act as neutrophil chemoattractants and are also essential in IRI (29). Although no consistent trends were observed at 2 hours after reperfusion, anti-Properdin-Ab significantly reduced the expression of TNF-α (**Figure 3B**). The expressions of CXCL-1 and -2 were significantly higher in the anti-Properdin-Ab group. At 6 hours, anti-Properdin-Ab significantly decreased the expressions of IL-1β and TNF-α.

### 3.5 Altered populations in liver-resident and infiltrating macrophages after hepatic IRI

**Figure 4:**
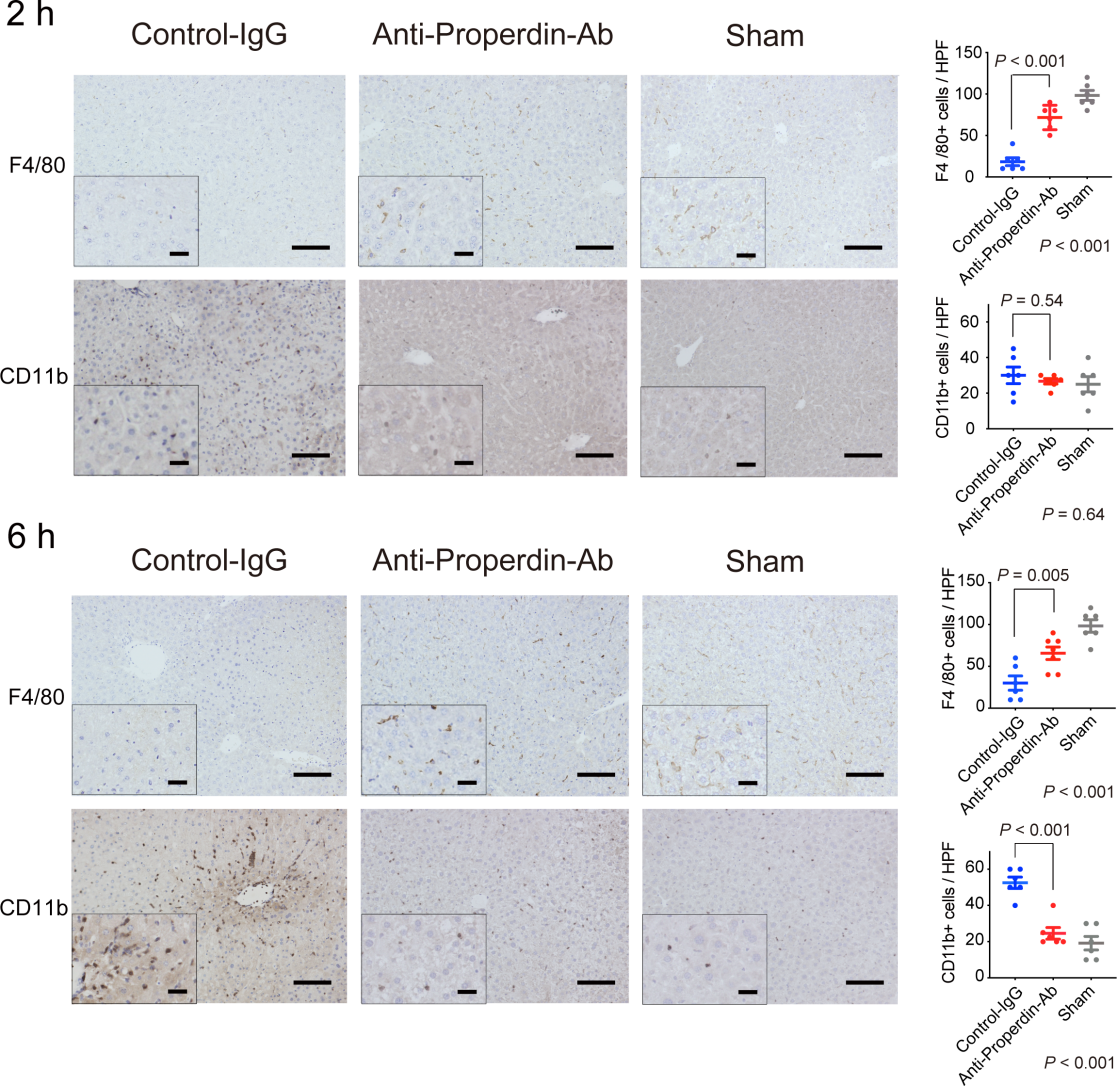
Alteration of macrophage populations by hepatic IRI. Immunohistochemical staining for F4/80+ and CD11b+ cells (*n* = 6 mice/group, magnification, ×200 [x400 for insets], Scale bars: 100 μm) and their quantification using ImageJ. The number of F4/80+ cell was significantly lower in the control group than in anti-Properdin-Ab group at 2 and 6 hours after reperfusion (*P <*0.001). The recruitment of CD11b+ cells in the control group did not significantly differ from that in the sham group at 2 h; however, they were recruited thereafter, and significantly more CD11b+ cells were evident in the control group than in the sham group at 6 h (*P <*0.001). The infiltration was significantly ameliorated in anti-Properdin-Ab group. Data from sham controls are shown as a reference. IgG, immunoglobulin; Ab, antibody.

Macrophages also play key roles in the pathogenesis of hepatic IRI (30–32), and the complement system has been shown to be a potent macrophage chemoattractant (6). To investigate the macrophage compositions in liver tissues after hepatic IRI, we conducted immunohistochemical staining for F4/80 and CD11b. F4/80 is a representative marker for macrophages, mainly Kupffer cells, while CD11b is for infiltrating macrophages (31, 33). As shown in **Figure 4**, hepatic IRI significantly lowered F4/80+ cells in liver tissues (Sham *vs.* Control, *P <*0.001), but these alterations were significantly attenuated by anti-Properdin-Ab at both 2 and 6 hours after reperfusion.

The number of CD11b+ cells in the control group did not significantly differ from that in the sham group at 2 hours after reperfusion; however, they were recruited thereafter, and significantly more CD11b+ cells were evident in the control group than in the sham group in the late phase (6 hours, *P <*0.001). This inflammatory cell infiltration was significantly ameliorated in anti-Properdin-Ab group. Liver macrophage subsets are characteristically altered by hepatic IR, i.e., decreases in Kupffer cells (KC) and increases in infiltrating macrophages (31). These deleterious alterations were significantly attenuated by anti-Properdin-Ab.

### 3.6 Heme oxygenase 1 expressions

**Figure 5:**
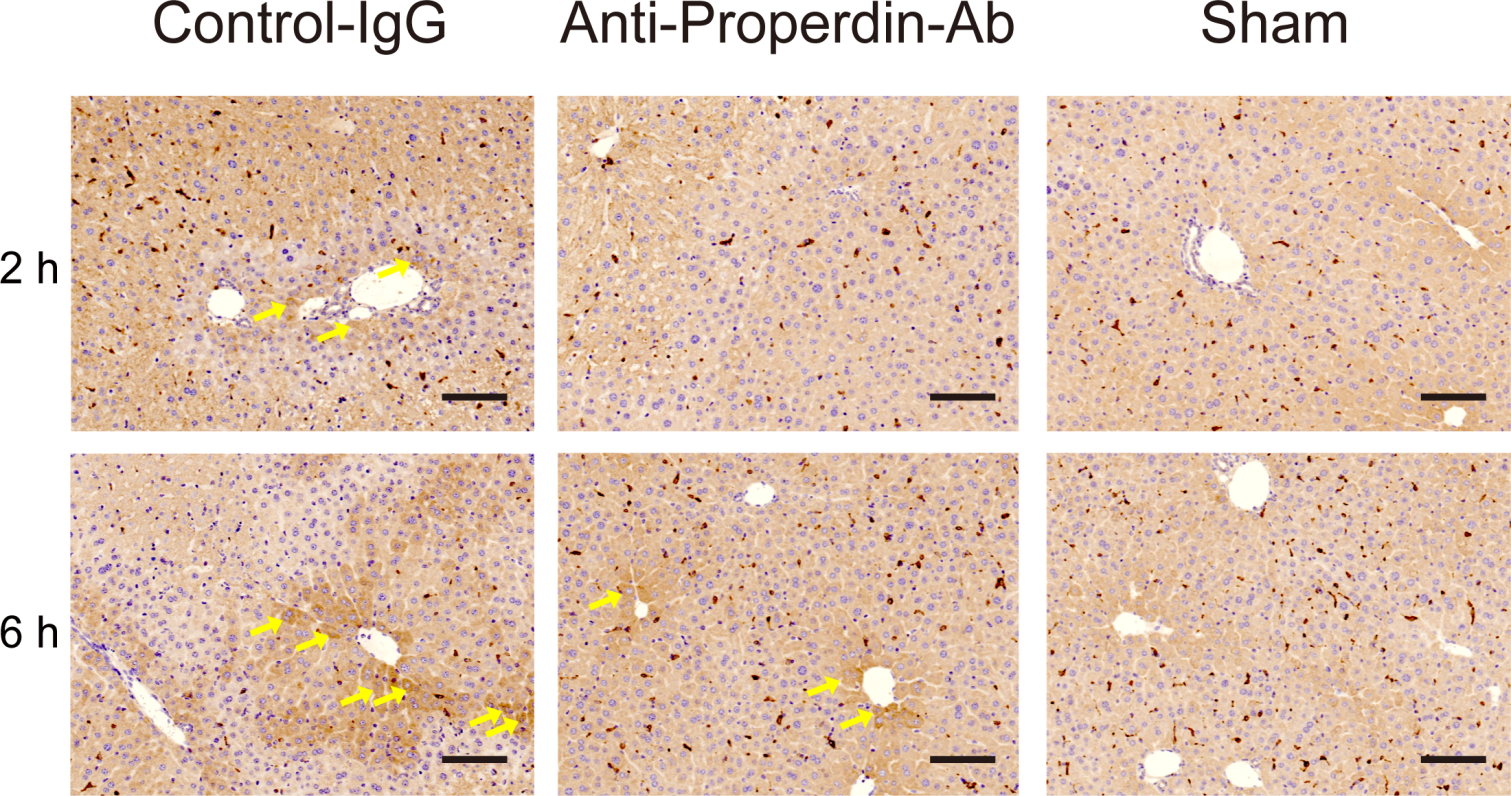
HO-1 expressions. Representative liver sections stained with the oxidative/inflammatory marker, HO-1, at 2 and 6 hours (*n* = 6 mice/group, magnification, ×200, Scale bar: 100 μm). HO-1 was diffusely expressed in macrophages in each group. HO-1 positive hepatocytes (arrows) were sporadically observed around portal (zone-1) and central venules (zone-3) in the control group at 2 hours. In contrast, they were rarely detected in anti-Properdin-Ab group. At 6 hours, densely stained HO-1 positive hepatocytes (arrows) were observed around zone-1 and -3 in the control group, while they were sporadically identified in anti-Properdin-Ab group. IgG, immunoglobulin; Ab, antibody.

HO-1, a rate-limiting enzyme catalyzing the conversion of heme into biliverdin, carbon monoxide, and ferrous iron, exerts anti-oxidative, cytoprotective, and anti-inflammatory functions (34). Therapeutic potential of HO-1 modulations against IR-stress in liver has been reported (35, 36). HO-1 was diffusely expressed in macrophages in each group (**Figure 5**). HO-1 positive hepatocytes were sporadically observed around portal (zone-1) and central venules (zone-3) in the control group at 2 hours after reperfusion, whereas they were rarely detected in anti-Properdin-Ab group. In the late phase (6hours), dense HO-1 staining was observed around zone-1 and -3 in the controls, while this was not detected in anti-Properdin-Ab group.

### 3.7 Neutrophil infiltration

**Figure 6:**
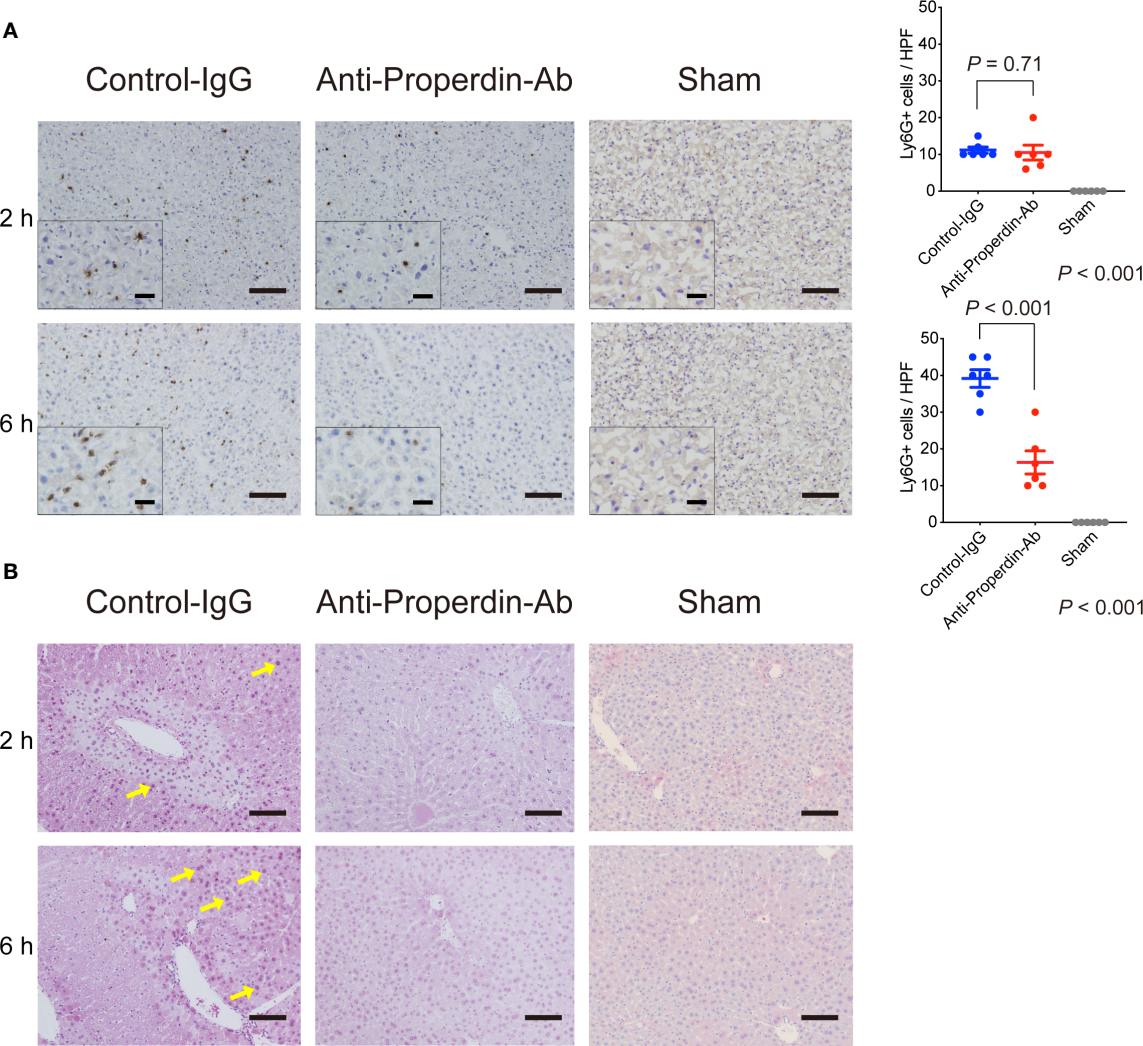
Neutrophil infiltration and oxidative stress. **(A)** Immunohistochemical staining for Ly6-G at 2 and 6 hours after reperfusion (*n* = 6 mice/group, magnification, ×200 [x400 for insets], Scale bars: 100 μm) and their quantification using ImageJ. Ly6G+ cells were not yet recruited remarkably at 2 h in both groups. The number of Ly6-G+ cells was significantly lower in anti-Properdin-Ab group than in the control group at 6 h. Data from sham controls are shown as a reference. **(B)** Representative liver sections stained with the oxidative stress marker, 8-OHdG, at 2 and 6 hours (*n* = 6 mice/group, magnification, ×200, Scale bar: 100 μm). Staining intensity of 8-OHdG was high in nuclei with severe DNA damage (yellow arrows), which was observed around portal (zone-1) or central venules (zone-3) in 2 out of 6 slides in the control group at 2 h. 8-OHdG+ cells were detected only sporadically in anti-Properdin-Ab group. At 6 hours, all slides in the control group showed diffusely-scattered positive hepatocytes panlobularly (yellow arrows). In contrast, positive cells were sporadically observed in some slides in anti-Properdin-Ab group, indicating that anti-Properdin-Ab significantly alleviated oxidative cell damage. IgG, immunoglobulin; Ab, antibody.

Properdin indirectly promotes C5aR-mediated neutrophil activation (13, 15), resulting in inflammatory responses and following tissue injury during hepatic IR (37). Ly6G+ cells, i.e., activated neutrophils, were not yet recruited remarkably at 2 hours after reperfusion; however, they significantly infiltrated thereafter in the late phase. These inflammatory responses were significantly alleviated by anti-Properdin-Ab compared with the control at 6 hours after reperfusion (**Figure 6A**).

### 3.8 Oxidative tissue damage

Reactive oxygen species (ROS) and resultant oxidative stress are also central in the pathogenesis of hepatic IRI. 8-OHdG, a sensitive marker for oxidative DNA damage, positive cells were clearly observed around portal (zone-1) and central venules (zone-3) in 2 out of 6 slides in the control group at 2 hours after reperfusion (**Figure 6B**), whereas they were detected only sporadically in anti-Properdin-Ab group. In the late phase (6 hours), dense 8-OHdG staining was observed panlobularly in the controls, while these oxidative cell damages were significantly alleviated by anti-Properdin-Ab administration.

### 3.9 Platelet aggregation in the hepatic microcirculation

**Figure 7:**
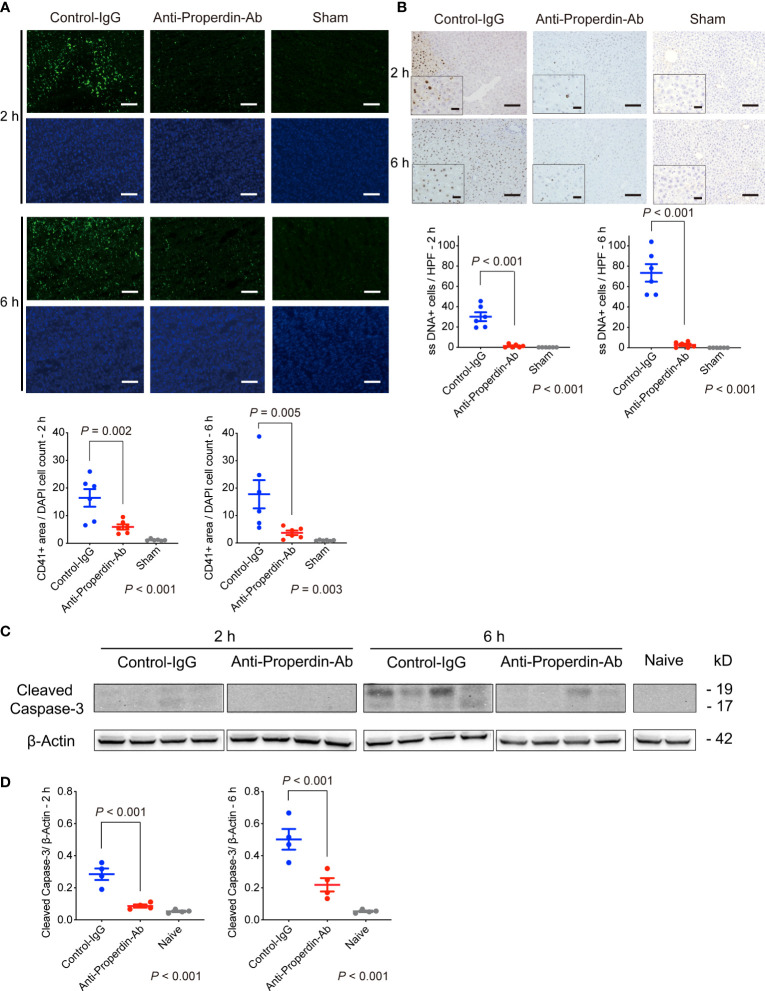
Platelet aggregation and apoptosis. **(A)** Representative tissue sections stained for CD41 (*n* = 6 mice/group, magnification, ×200, Scale bar: 100 μm), and their quantification using ImageJ. Platelet thrombi, represented by CD41+ spots, were significantly fewer in anti-Properdin-Ab group than in the control group at 2 and 6 hours after reperfusion. Data from sham controls are shown as a reference. **(B)** Representative ssDNA staining of liver sections (*n* = 6 mice/group, magnification, ×200 [x400 for insets], Scale bars: 100 μm) and its quantification (ImageJ). The number of ssDNA-positive cells was significantly lower in anti-Properdin-Ab group at both 2 and 6 hours after reperfusion. Data from sham controls are shown as a reference. **(C)** The expression of cleaved caspase-3 at 6 h after reperfusion was significantly weaker in anti-Properdin-Ab group than in the control group (*n* = 4 mice/group). **(D)** Protein expression levels were quantified using ImageJ and normalized to those of β‐actin. Data from naive controls are shown as a reference. IgG, immunoglobulin; Ab, antibody.

Platelet aggregation and subsequent microcirculatory impairment are one of the main pathological features underlying hepatic IRI (38), which is enhanced by complement activation (39) (40). Platelet thrombi, represented by CD41+ spots, were significantly fewer in anti-Properdin-Ab group than in the control group at both 2 and 6 hours after reperfusion (**Figure 7A**). Thus, platelet aggregation started early after hepatic IR, but it was significantly attenuated by anti-Properdin-Ab.

### 3.10 Cell apoptosis

Since apoptosis is one of the main forms of cell death in IRI (2), we examined apoptosis induced by IR. As shown in **Figure 7B**, ssDNA-positive cells were significantly increased after hepatic IRI, which was significantly attenuated by anti-Properdin-Ab (*vs*. Control, *P <*0.001 at 2 and 6 hours, respectively). Western blot analysis also demonstrated significant up-regulation of cleaved caspase-3 after hepatic IRI, which was significantly alleviated by anti-Properdin-Ab (*vs*. Control, *P <*0.001 at 2 and 6 hours, respectively, **Figures 7C, D**).

### 3.11 Analysis of signal pathways in western blot

**Figure 8:**
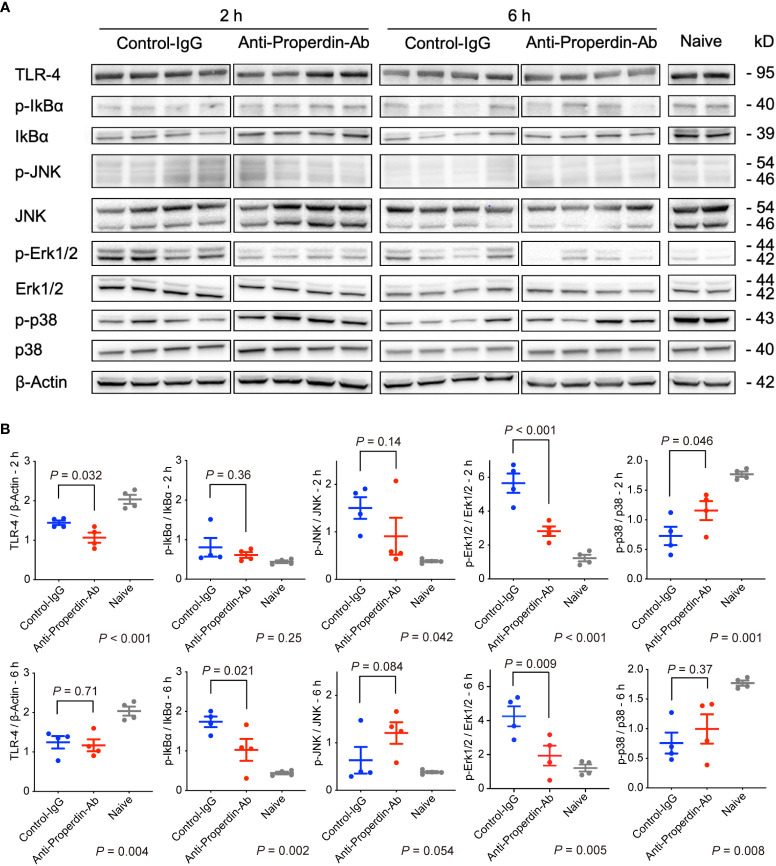
Western blot for upstream signals. **(A)** Anti-Properdin-Ab downregulated only Erk1/2 activation at both time points (*n* = 4 mice/group). **(B)** Protein expression levels were quantified using ImageJ. Data from naive controls are shown as a reference. IgG, immunoglobulin; Ab, antibody.

Mitogen-activated protein kinases (MAPKs) including c-jun N-terminal Kinase (JNK), p-38, and extracellular signal-regulated kinase 1/2 (Erk1/2), are closely involved in IRI (41, 42) and apoptosis (43, 44). Therefore, we comprehensively assessed various upstream signaling pathways leading to hepatic tissue injury or apoptosis using Western blot. As shown in **Figures 8A, B**, only Erk1/2 up-regulation was significantly attenuated by anti-Properdin-Ab treatment at both 2 and 6 hours.

### 3.12 The influence of anti-Properdin-Ab on liver regeneration after partial hepatectomy

**Figure 9:**
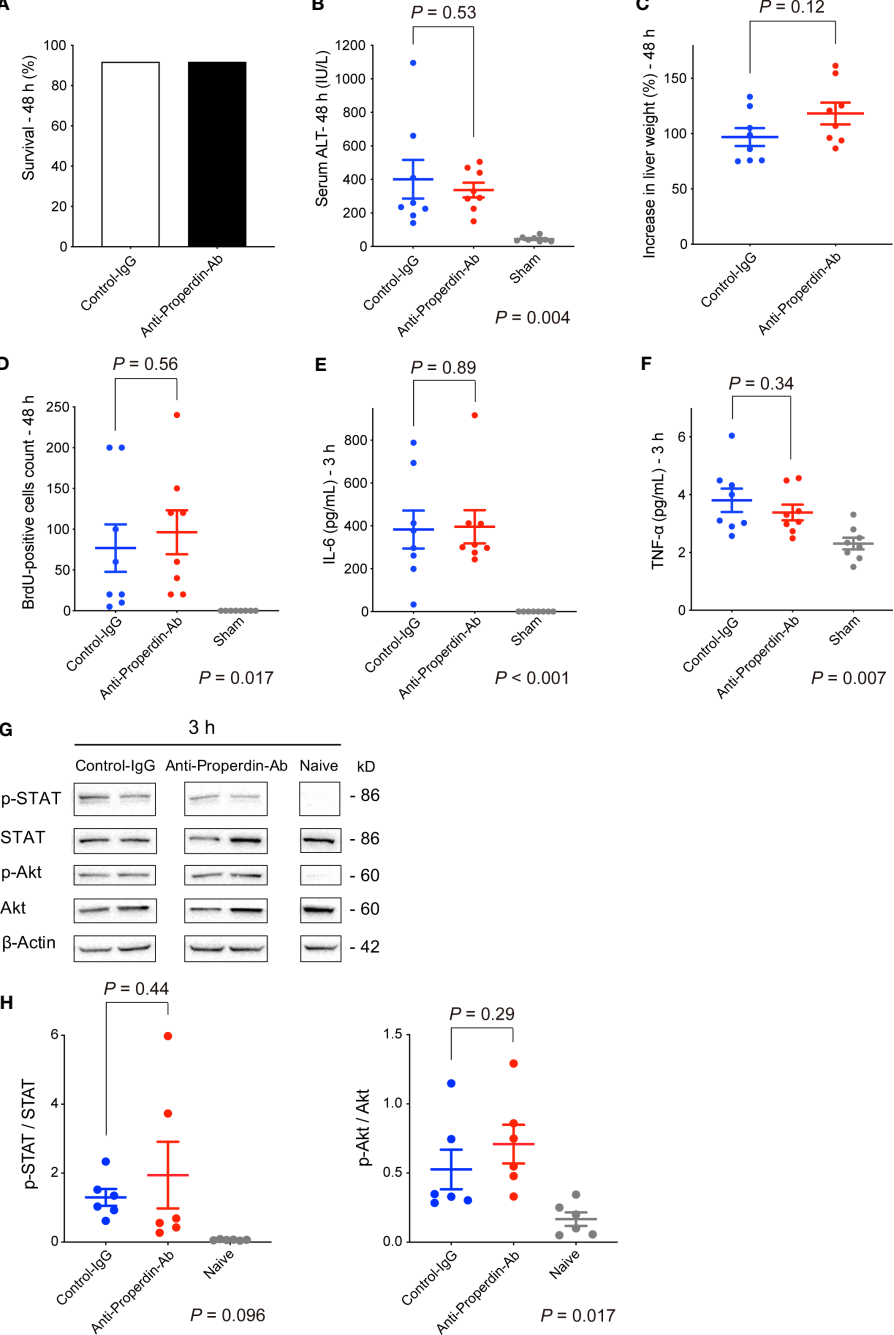
Liver regeneration in 70% partial hepatectomy. Ten out of twelve mice in each group survived for more than 48 hours after hepatectomy **(A)**. No significant differences were observed in serum ALT **(B)**, increase in liver weight **(C)**, and BrdU-positive cell count **(D)** at 48 hours after 70% hepatectomy (*n* = 8 mice/group). There were no significant differences in serum IL-6 **(E)** and TNF-α **(F)** levels at 3 hours after hepatectomy between the two groups (*n* = 8 mice/group). **(G)** Phosphorylation of STAT and Akt in the remnant liver tissues was similar between the two groups at 3 hours. **(H)** Protein expression levels were quantified using ImageJ and normalized to those of STAT and Akt (*n* = 6 mice/group). Data from sham and naïve controls are shown as a reference **(B-E, G, H)**. IgG, immunoglobulin; Ab, antibody.

Well-known anaphylatoxins, C3a and C5a, play pivotal roles in liver regeneration (45); therefore, the influence of Properdin inhibition on liver regeneration was evaluated using a mouse model of 70% partial hepatectomy. Ten out of twelve mice survived for 48 hours after hepatectomy in each group (**Figure 9A**) with no difference in serum ALT at 48 h (**Figure 9B**). In terms of liver regeneration, there was no significant difference in both liver-weight increase and the number of BrdU-positive cells at 48 h (**Figures 9C, D**, respectively).

Since IL-6 and TNF-α levels are essential especially in the priming phase of the regenerative response (21, 46), their serum concentrations were both measured at 3 hours after surgery. No significant differences were observed between the control and the anti-Properdin-Ab group (**Figures 9E, F**, respectively).

Both signal transducer and activator of transcription (STAT) and protein kinase B (Akt) are important transcription factors in the proliferative signaling pathways after hepatectomy (21, 46). Their phosphorylation in remnant livers was similar between the two groups (**Figures 9G, H**).

## 4 Discussion

The complement system plays a critical role in hepatic IRI (6, 7); however, no complement-targeted treatments have been introduced in a clinical setting. In the present study, we focused on Properdin, the only known positive regulator of the AP, and demonstrated significant therapeutic effects of Properdin inhibition on hepatic IRI: Anti-Properdin-Ab administration resulted in attenuation of platelet aggregation in hepatic microcirculation, decreased hepatocyte apoptosis, maintained macrophage subsets, and reduced neutrophil activation with ROS generation. Moreover, Properdin inhibition did not affect liver regeneration negatively after 70% partial hepatectomy. These results highlight the therapeutic potential of anti-Properdin-Ab in liver resection and liver transplantation.

It has been reported that AP plays an important role in cerebral, renal, and myocardial IRI (47–49); however, its association with hepatic IRI remains to be elucidated. Importantly, AP amplifies C3 activation in the complement system (50), in which Properdin stabilizes the C3 convertases that cleaves C3 into C3a and C3b, prolonging its half-life by 5-10 fold. C3a is a well-known potent anaphylatoxin, and its receptor (C3aR) are expressed on a variety of inflammatory cells including monocytes and neutrophils (50, 51). The C3a-C3aR interaction is a strong immunomodulator/chemoattractant that recruits immune cells to the site of activation. Therefore, the reduction of C3 cleavage by anti-Properdin-Ab should partly explain the amelioration of hepatic IRI. In fact, sCR-1, which efficiently reduces C3 activation, alleviated hepatic IRI by maintaining microvascular circulation and reducing adherent leukocytes (9).

Since complement activation by AP accounts for approximately 80% of terminal pathway activity (13), we assumed that the inhibition of C5 cleavage by anti-Properdin-Ab also significantly contributed to the suppression of hepatic IRI. Properdin accelerates C3 cleavage, and subsequent C3b upregulation leads to the activation of C5 convertases, which cleave C5 into C5a, a potent chemoattractant (52), and C5b, a component of the membrane attack complex (MAC) (53). Similar to C3aR, C5a receptors (C5aR) have been identified on various immune/inflammatory cells (50, 51). In hepatic IRI, C5a-C5aR interaction provokes leukocyte activation and transmigration, thereby causing hepatocyte necrosis/apoptosis (10, 12). MAC directly lyses target cells and facilitates hepatocyte apoptosis, enhancing the release of DAMPs, cytokines/chemokines, and other anaphylatoxins (11, 21, 54). We recently reported the therapeutic potential of anti-C5 antibody in hepatic IRI (12). Of interest, the mechanisms underlying its protective effects, e.g., ameliorating platelet aggregation, hepatocyte apoptosis, and macrophages/neutrophils infiltration, *etc.*, were also observed in the current study, implying that Properdin inhibition may also exert its protective effects by inhibiting the terminal pathway.

A recent study claimed that hepatic IR resulted in the necrotic reduction/depletion of liver-resident macrophages, Kupffer cells (F4/80+, CD11b-), while monocyte-derived macrophages (F4/80+, CD11b+) transmigrated to the liver alternatively (31). In the present study, similar alterations were observed in the control group, which were, interestingly, suppressed by anti-Properdin-Ab. Maintained physiological populations of liver macrophages in the treatment group may be partly attributable to decreased C3a-C3aR- and C5a-C5aR mediated signals by anti-Properdin-Ab. However, this finding may simply be the result of reduced hepatic injury. Further studies are warranted to elucidate the mechanisms underlying the altered macrophage response. In addition, our study did not discriminate Kupffer cells from monocyte-derived macrophages. We attempted double immunofluorescence staining of F4/80 and CD11b; however, F4/80+/CD11b+ cells were not visualized (data not shown) and the alteration of these two subsets of macrophages was not accurately assessed.

It is also noteworthy that Properdin inhibition significantly reduced the formation of platelet thrombi from the early phase of reperfusion. Needless to say, microcirculatory disturbance is detrimental in hepatic IRI, as energy supply becomes insufficient and ATP restoration is impaired upon reperfusion (2). Thus, maintained microcirculation undoubtedly contributes to alleviating hepatic IRI. C3a-C3aR interaction activates platelets, and insertion of MAC through the platelet membrane promotes the release of prothrombotic platelet micro-vesicles (50). C5a-C5aR interaction on monocytes and endothelial cells induces up-regulation of tissue factor, promoting coagulation through the extrinsic pathway. It is likely that these reactions were indirectly suppressed by anti-Properdin-Ab in our study.

MAPKs including JNK, p-38, and Erk1/2 play pivotal roles in provoking IRI (41, 42) and apoptosis (43, 44). In our study, only Erk1/2 activation was alleviated by Properdin inhibition. Erk is responsible for various cellular activities, such as proliferation, differentiation, and apoptosis (41, 43). For example, Erk is reported to be capable of promoting intrinsic or extrinsic apoptotic pathways by inducing caspase-8 activation or mitochondrial cytochrome c release (43). This may partly explain the reason that Properdin inhibition significantly reduced hepatocyte apoptosis. Regarding the association between the complement system and Erk signaling, anaphylatoxin C5a-C5aR interaction induces chemotaxis/activation of macrophages and their cytokine release by stimulating MAPK cascades (55, 56). It is possible that Properdin inhibition suppresses a series of the reaction by reducing C5a production and subsequent Erk1/2 activation.

Impaired liver regeneration is closely associated with the degree of hepatic IRI (2, 3). Thus, reducing hepatic IRI may promote sufficient restoration of the remnant liver after liver resection or partial liver transplantation. However, complement inhibition, while effective in ameliorating hepatic IRI, has been reported to negatively affect liver regeneration. C3a and C5a are potent anaphylatoxins and contribute substantially to initiating hepatocyte proliferation (45, 57). Complete inhibition of these molecules would result in reduced IL-6 and TNF levels, thereby inactivating the STAT3 and PI3K/Akt pathways, both of which are necessary for liver regeneration (57). We recently reported significant protection by anti-C5 antibody for hepatic IRI in a rodent model (12); however, complete C5 inhibition raises a concern for inadequate liver regeneration after extensive hepatectomy and partial liver transplantation (19). The present study revealed that Properdin inhibition did not affect liver regeneration even after 70% partial hepatectomy. This may be due to its only partial inhibition of C3a and C5a production by Properdin inhibition. Consistently, a previous study claimed that liver regeneration was not impaired after partial hepatectomy if C3 production was partially/appropriately blocked (46). C3a and C5a are essential for liver regrowth; however, their excessive production may disrupt the delicate balance between liver injury and regeneration.

In conclusion, anti-Properdin-Ab significantly attenuated hepatic IRI without compromising liver regeneration. To date, complement-targeted interventions have yet to be applied into clinical practice. Based on its efficacy and safety, Properdin inhibition may provide a novel therapeutic strategy for liver resection and liver transplantation.

## Data availability statement

The raw data supporting the conclusions of this article will be made available by the authors, without undue reservation.

## Ethics statement

The animal study was reviewed and approved by the Animal Research Committee of Kyoto University (MedKyo-17546 and -18193).

## Author contributions

JK and KH conceived and designed the study. JK, TTa, and HM participated in the performance of the research. JK, KH, TTa, HM, XZ, SK, and TTs participated in the analysis and interpretation of data. JK and KH wrote the manuscript. EH edited the final version of the draft. KH obtained the research grant. All authors contributed to the article and approved the submitted version.
