# Supplementary material for: Properdin inhibition ameliorates hepatic ischemia/reperfusion injury without interfering with liver regeneration in mice
Source: Front Immunol. 2023 Aug 16;14:1174243. doi: 10.3389/fimmu.2023.1174243 (PMC10469474; doi:10.3389/fimmu.2023.1174243)
Supplement: Supplementary file 1 [file Table_1.docx]

Supplementary Material

Properdin inhibition ameliorates hepatic ischemia/reperfusion injury without interfering with liver regeneration in mice.

**Jiro Kusakabe, MD, PhD, MPH^1^, Koichiro Hata, MD, PhD^1^**^*^**, Tetsuya Tajima, MD, PhD, MPH^1^, Hidetaka Miyauchi, MD, PhD^1^, Xiangdong Zhao, MD, PhD^1^, Shoichi Kageyama, MD, PhD^1^, Tatsuaki Tsuruyama, MD, PhD^2^, and Etsuro Hatano, MD, PhD^1^**

^1.^ Department of Surgery, Division of Hepato-Biliary-Pancreatic Surgery and Transplantation, Graduate School of Medicine, Kyoto University, Kyoto, Japan

^2.^ Center for Anatomical, Pathological, and Forensic Medical Research, Graduate School of Medicine, Kyoto University, Kyoto, Japan

*** Correspondence:**Koichiro Hata, M.D., Ph.D.

Division of Hepato-Biliary-Pancreatic Surgery and Transplantation, Department of Surgery, Graduate School of Medicine, Kyoto University

54 Kawahara-cho, Shogoin, Sakyo-ku, 606-8507 Kyoto, Japan

Tel.: +81-75-751-4323, Fax: +81-75-751-4348

E-mail: khata@kuhp.kyoto-u.ac.jp

**Supplemental Table 1**

Primer sequences used in the present study.

|  | Primer sequences (5'-3') | |
| --- | --- | --- |
| Genes | Forward | Reverse |
| GAPDH | TGTGTCCGTCGTGGATCTGA | TTGCTGTTGAAGTCGCAGGAG |
| IL-1β | GGTCAAAGGTTTGGAAGCAG | TGTGAAATGCCACCTTTTGA |
| IL-6 | ACCAGAGGAAATTTTCAATAGGC | TGATGCACTTGCAGAAAACA |
| TNF-α | AGGGTCTGGGCCATAGAACT | CCACCACGCTCTTCTGTCTAC |
| CXCL-1 | TCTCCGTTACTTGGGGACAC | CCACACTCAAGAATGGTCGC |
| CXCL-2 | TCCAGGTCAGTTAGCCTTGC | CGGTCAAAAAGTTTGCCTTG |

**Supplemental Table 2**

Primary antibodies used in immunohistochemistry.

| Antibody | Target | Species | Company | Catalog No. | Dilution |
| --- | --- | --- | --- | --- | --- |
| C3 | Complement 3 activation | Rabbit polyclonal | Abcam, Cambridge, UK | ab11887 | 1:200 |
| F4/80 | Macrophages | Rat monoclonal | eBioscience, San Diego, CA | 14-4801 | 1:50 |
| 8-hydroxy-2’-deoxyguanosine  (8-OHdG) | Oxidative stress | Mouse monoclonal | Japan Institute for the Control of Aging, Nikken SEIL, Shizuoka, Japan | clone N45.1 | 1:10 |
| CD11b | Infiltrating macrophages/ neutrophils | Rabbit polyclonal | Abcam, Cambridge, UK | ab75476 | 1:400 |
| single-stranded DNA (ssDNA) | Apoptotic cell death | Rabbit polyclonal | IBL, Fujioka, Japan | 18731 | 1:600 |
| CD41* | Platelet activation/ aggregation | Rat monoclonal | GeneTex, Irvine, CA | MWReg30 | 1:100 |
| Lymphocyte antigen 6 complex locus G (Ly6-G)* | Neutrophils | Rat monoclonal | Tonbo Biosciences, Irvine, CA | 5931 | 1:10 |
| BrdU | DNA synthesis | Mouse monoclonal | Leica Biosystems, Buffalo, IL | NCL-BrdU | 1:400 |
| Heme oxygenase 1 (HO-1) | Macrophages, hepatocytes | Rabbit polyclonal | Proteintech Group, IL | 10701-1-AP | 1:200 |

* Antibodies were applied to frozen liver sections.

**Supplemental Table 3**

| Antibody | Species | Company | Catalog No. | Dilution |
| --- | --- | --- | --- | --- |
| β-Actin | Rabbit polyclonal | MBL, Nagoya, Japan | PM053 | 1:2000 |
| Cleaved Caspase-3 | Rabbit polyclonal | AST | 9661 | 1:500 |
| TLR-4 | Rabbit polyclonal | Novus Biologicals, Littleton, CO | NB100-56581 | 1:500 |
| p-IkBα | Rabbit monoclonal | CST | 2859 | 1:1000 |
| p-JNK | Rabbit monoclonal | CST | 4671 | 1:1000 |
| p-ERK1/2 | Rabbit monoclonal | CST | 4370 | 1:2000 |
| p-p38 | Rabbit monoclonal | CST | 4511 | 1:1000 |
| p-STAT | Rabbit monoclonal | CST | 9145 | 1:2000 |
| p-Akt | Rabbit monoclonal | CST | 4060 | 1:2000 |
| IkBα | Rabbit monoclonal | CST | 4812 | 1:1000 |
| JNK | Rabbit polyclonal | CST | 9252 | 1:1000 |
| ERK1/2 | Rabbit monoclonal | CST | 4695 | 1:1000 |
| p38 | Rabbit monoclonal | CST | 8690 | 1:1000 |
| STAT | Rabbit monoclonal | CST | 12640 | 1:1000 |
| Akt | Rabbit monoclonal | CST | 4691 | 1:1000 |
